# Social Disengagement and Cognitive Function: Does the Association Vary by Gender?

**DOI:** 10.1093/geroni/igab046.2614

**Published:** 2021-12-17

**Authors:** Bada Kang, Eunhee Cho, Sarah Oh

**Affiliations:** 1 Mo-Im Kim Nursing Research Institute, Yonsei University College of Nursing, Seoul, Seoul-t'ukpyolsi, Republic of Korea; 2 Yonsei University College of Nursing, Seoul, Seoul-t'ukpyolsi, Republic of Korea

## Abstract

Although social disengagement is considered to be a predictor of cognitive decline, and increase risk of Alzheimer’s and related dementias, little is known regarding the gender-specific association between social disengagement and cognition among Korean middle-aged and older adults. Korea’s Confucianism-based gender roles provide unique contexts to examine gender differences in the influence of social disengagement on cognition. This study investigated the association between social disengagement and cognitive function in a nationally representative sample of Koreans aged 45 years or older (N = 5,196 women and 2,707 men), using data from the Korean Longitudinal Study of Aging (2008-2018). Results from the generalized estimating equation model showed that compared to consistent social engagement, consistent non-engagement was significantly associated with lower cognitive function among both genders. Transitioning from social engagement to non-engagement was significant for males only. Of various types of social activities (religious, senior center, sport, reunion, voluntary, political), consistent non-engagement in a senior center was most associated with lower cognitive function among both genders, while consistent non-engagement in religious activities was significant for females only. While household arrangements were not associated with cognition in men, widowed women had increased risk of cognitive decline than married women, as did women living in households of three or more people. Depression was a predictor of cognitive decline among males only. In this gender-specific study, we found that consistent participation in social activities, especially via membership in a senior community center, is beneficial in preventing cognitive decline among both genders.

